# Mol­ecular structures of the penta­phenyl­cyclo­penta­dienyl iron com­plexes [(C_5_Ph_5_)Fe(CO)_2_ *R*] (*R* = Me, Ph, iPr and Bu)

**DOI:** 10.1107/S2053229621006057

**Published:** 2021-06-16

**Authors:** Karlheinz Sünkel, Christian Klein-Hessling

**Affiliations:** a Chemistry, Ludwig-Maximilians-University Munich, Butenandtstrasse 9, Munich D-81377, Germany

**Keywords:** penta­phenyl­cyclo­penta­dien­yl, iron, carbon­yl, Pd-catalysed, crystal structure

## Abstract

The Pd^II^-catalysed reaction of [(C_5_Ph_5_)Fe(CO)_2_Br] with Grignard com­pounds *R*Mg*X* or butyl lithium gave the iron alk­yl/aryl com­plexes [(C_5_Ph_5_)Fe(CO)_2_ *R*] (*R* = Me, Ph, iPr and Bu) in 59–73% yield, namely, di­carbonyl­meth­yl(η^5^-penta­phenyl­cyclo­penta­dien­yl)iron, [Fe(CH_3_)(C_35_H_25_)(CO)_2_], dicarbon­yl(η^5^-penta­phenyl­cyclo­penta­dien­yl)phenyliron, [Fe(C_6_H_5_)(C_35_H_25_)(CO)_2_], dicarbon­yl(isoprop­yl)(η^5^-penta­phenyl­cyclo­penta­dien­yl)iron, [Fe(C_3_H_7_)(C_35_H_25_)(CO)_2_], and butyl­dicarbon­yl(η^5^-penta­phenyl­cyclo­penta­dien­yl)iron, [Fe(C_4_H_9_)(C_35_H_25_)(CO)_2_]. The crystal structure determinations showed the usual ‘paddle-wheel’ orientation of the phenyl rings, with an average canting angle of *ca* 50°. The bond parameters are mainly dictated by the steric requirements of the alk­yl/aryl groups and only the phenyl com­plex shows electronic effects.

## Abstract 2

The penta­phenyl­cyclo­penta­dienyl iron alkyl and aryl com­plexes [(C_5_Ph_5_)Fe(CO)_2_ *R*] (*R* = Me, Ph, iPr and Bu) were prepared and their crystal structures examined.

## Introduction   

Penta­aryl­cyclo­penta­dienyl com­plexes have been known for nearly 60 years. They were studied mainly because the bulky nature of these cyclo­penta­dienyl derivatives gives their com­plexes high kinetic stability, including the formation of stable radicals (Field *et al.*, 2011 ▸) or unusual structures in main group or lanthanoid metallocenes (Schulte *et al.*, 2020 ▸). Also, several examples of their application as asymmetric catalysts (Ruble *et al.*, 1997 ▸; Field *et al.*, 2011 ▸) and as mimics for hydrogenase (Hemming *et al.*, 2018 ▸) were found. So far (*Scifinder*, accessed on 12th May 2021), 451 publications describing 723 substances have appeared, an increase of 80% during the last decade. On the other hand, a survey of the Cambridge Structural Database (CSD, Version 5.42, accessed on 5th June, 2021; Groom *et al.*, 2016 ▸) showed only 118 entries, of which roughly half (52) contained iron as the central metal atom. Of these, *ca* 80% (41) were ferrocene derivatives. The mol­ecular structure of the very first penta­phenyl­cyclo­penta­dienyl com­plex, *i.e.* [(C_5_Ph_5_)Fe(CO)_2_Br] (McVey & Pauson, 1965 ▸), was published only 25 years later (Field *et al.*, 1989 ▸) and there are only three other structure determinations of mol­ecules containing the [(C_5_Ph_5_)Fe(CO)_2_] moiety in the CSD: MARFET and MARFIX (Hemming *et al.*, 2018 ▸), and PUYDES (Carter *et al.*, 2002 ▸). A very important subgroup of com­pounds containing the [CpFe(CO)] moiety contains the derived alkyl and aryl com­plexes [CpFe(CO)_2_ *R*] (Pannell & Sharma, 2010 ▸). These com­pounds were shown to have catalytic properties, for example, in de­hydrogenative couplings (Fukumoto *et al.*, 2015 ▸; Argouarch *et al.*, 2012 ▸) or, perhaps more importantly, as reagents in photoinduced DNA cleavage (Mohler *et al.*, 2002 ▸; Mohler & Shell, 2005 ▸). Therefore, it seemed worthwhile to study com­pounds of the type [(C_5_Ph_5_)Fe(CO)_2_ *R*], which might combine the unique properties of the penta­phenyl­cyclo­penta­dienyl moiety with the reactivity of the iron–alkyl and iron–aryl groups. Such com­pounds have been reported before, but were usually only partially characterized (Connelly & Manners, 1989 ▸; Brégaint *et al.*, 1990 ▸, 1992 ▸; Kuksis & Baird, 1994 ▸; Kuksis *et al.*, 1996 ▸). In particular, no crystal structures have been published. During the course of our studies on the coordination chemistry of perhalogenated cyclo­penta­dienyl com­plexes (Klein-Heßling *et al.*, 2021 ▸; Sünkel *et al.*, 2015 ▸) we also studied the [(C_5_ *X*_5_)Fe(CO)_2_ *R*] system. In the search for possible synthetic applications for these com­pounds and also for the sake of com­parison, we chose to prepare the alkyl and aryl [(C_5_Ph_5_)Fe(CO)_2_ *R*] derivatives (*R* = Me, **1**, Ph, **2**, iPr, **3**, and Bu, **4**) (Scheme 1). We report here the results of our crystal structure studies. 
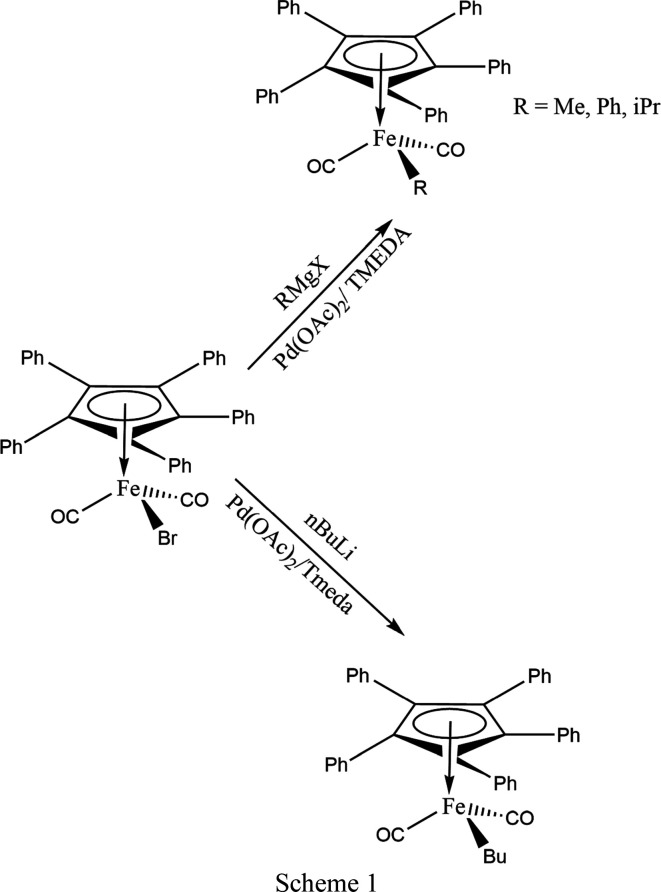


## Experimental   

### Synthesis and crystallization   

The starting material [(C_5_Ph_5_)Fe(CO)_2_Br] was prepared according to the literature from Fe(CO)_5_ and C_5_Ph_5_Br (McVey & Pauson, 1965 ▸). The reagents MeMgBr (3.0 *M* solution in Et_2_O), PhMgCl [2.0 *M* solution in tetra­hydro­furan (THF)], iPrMgCl (1.3 *M* solution with LiCl in THF) and BuLi (2.5 *M* solution in hexa­ne), as well as *N*,*N*,*N*′,*N*′-tetra­methyl­ethylendi­amine (TMEDA) and palladium acetate, were commercial products (Sigma–Aldrich) and were used as provided.

#### [(C_5_Ph_5_)Fe(CO)_2_Me], (1)   

A solution of [(C_5_Ph_5_)Fe(CO)_2_Br] (0.30 g, 0.47 mmol) in THF (12 ml), palladium(II) acetate (0.01 g, 0.05 mmol) and TMEDA (0.07 ml, 0.71 mmol) was treated at 0 °C with an MeMgBr solution (0.24 ml, 0.71 mmol) and stirred for 60 min. After evaporation of the solvent, the residue was redissolved in the minimum amount of petroleum ether and placed on top of a silica-gel chromatography column. Elution with petroleum ether/Et_2_O (9:1 *v*/*v*) yielded, after evaporation, com­pound **1** as a yellow solid (yield: 0.17 g, 0.30 mmol, 63%). Crystals suitable for X-ray diffraction were obtained by slow evaporation of a petroleum ether solution in a refrigerator at 5 °C.

IR (ATR): ν (CO) 1993, 1941 cm^−1^. UV–Vis (CH_2_Cl_2_): *λ*_max_ = 369 nm. ^1^H NMR (CDCl_3_, 400 MHz): δ 7.20–6.90 (*m*, Ph), 0.62 (*s*, Me) ppm. ^13^C NMR (CDCl_3_, 100.5 MHz): δ 217.8 (CO), 132.2, 131.9, 127.7, 127.5 (4 × Ph), 102.1 (C5), −7.5 (Me) ppm.

#### [(C_5_Ph_5_)Fe(CO)_2_Ph], (2)   

A solution of [(C_5_Ph_5_)Fe(CO)_2_Br] (0.05 g, 0.08 mmol) in THF (10 ml), palladium(II) ace­tate (0.002 g, 0.01 mmol) and TMEDA (0.01 ml, 0.08 mmol) was treated at 0 °C with a PhMgCl solution (0.05 ml, 0.10 mmol) and stirred for 60 min. After evaporation of the solvent, the residue was redissolved in the minimum amount of petroleum ether and placed on top of a silica-gel chromatography column. Elution with petroleum ether/Et_2_O (9:1 *v*/*v*) yielded, after evaporation, com­pound **2** as a yellow solid (yield: 0.03 g, 0.05 mmol, 59%). Crystals suitable for X-ray diffraction were obtained by slow evaporation of a petroleum ether solution in a refrigerator at 5 °C.

IR (ATR): ν (CO) 2009, 1968 cm^−1^. UV–Vis (CH_2_Cl_2_): λ_max_ = 364 nm. ^1^H NMR (CDCl_3_, 400 MHz): δ 7.23–6.81 (*m*, Ph) ppm. ^13^C NMR (CDCl_3_, 100.5 MHz): δ 216.5 (CO), 147–123 (35 × Ph), 102.8 (C5) ppm.

#### [(C_5_Ph_5_)Fe(CO)_2_iPr], (3)   

A solution of [(C_5_Ph_5_)Fe(CO)_2_Br] (0.10 g, 0.16 mmol) in THF (10 ml), palladium(II) acetate (0.004 g, 0.02 mmol) and TMEDA (0.02 ml, 0.16 mmol) was treated at 0°C with an iPrMgCl solution (0.18 ml, 0.24 mmol) and stirred for 60 min. After evaporation of the solvent, the residue was redissolved in the minimum amount of petroleum ether and placed on top of a silica-gel chromatography column. Elution with petroleum ether/Et_2_O (9:1 *v*/*v*) yielded, after evaporation, com­pound **3** as a yellow solid (yield: 0.07 g, 0.12 mmol, 73%). Crystals suitable for X-ray diffraction were obtained by slow evaporation of a petroleum ether solution in a refrigerator at 5 °C.

IR (ATR): ν (CO) 1991, 1939 cm^−1^. UV–Vis (CH_2_Cl_2_): λ_max_ = 367 nm. ^1^H NMR (CDCl_3_, 400 MHz): δ 7.32–6.85 (*m*, Ph), 3.07 (*m*, CHMe_2_), 1.46 (*m*, CHMe_2_) ppm.

#### [(C_5_Ph_5_)Fe(CO)_2_Bu], (4)   

A solution of [(C_5_Ph_5_)Fe(CO)_2_Br] (0.05 g, 0.08 mmol) in THF (10 ml), palladium(II) acetate (0.002 g, 0.01 mmol) and TMEDA (0.01 ml, 0.08 mmol) was treated at −30 °C with a BuLi solution (0.04 ml, 0.10 mmol) and stirred for 60 min. After evaporation of the solvent, the residue was redissolved in the minimum amount of petroleum ether and placed on top of a silica-gel chromatography column. Elution with petroleum ether/Et_2_O (9:1 *v*/*v*) yielded after evaporation com­pound **4** as a yellow solid (yield: 0.03 g, 0.05 mmol, 63%). Crystals suitable for X-ray diffraction were obtained by slow evaporation of a petroleum ether solution in a refrigerator at 5 °C.

IR (ATR): ν (CO) 1993, 1939 cm^−1^. UV–Vis (CH_2_Cl_2_): λ_max_ = 378 nm. ^1^H NMR (CDCl_3_, 400 MHz): δ 7.22–6.84 (*m*, Ph), 1.95–0.80 (4 *m*, Bu) ppm. ^13^C NMR (CDCl_3_, 100.5 MHz): δ 218.7 (CO), 132.2, 132.0, 127.7, 127.4 (4 × Ph), 102.3 (C5), 38.5, 28.2, 17.7, 14.1 (4 × Bu) ppm.

### Refinement   

Crystal data, data collection and structure refinement details are summarized in Table 1 ▸. H atoms on C atoms were calculated in ideal positions riding on their parent atoms, with C—H = 0.95 Å and *U*_iso_(H) = 1.2*U*_eq_(C) for aromatic H atoms, and C—H = 0.98 Å and *U*_iso_(H) = 1.5*U*_eq_(C) for methyl H atoms. The methyl groups were allowed to rotate along the C—C bonds to best fit the experimental electron density.

## Results and discussion   

The title com­pounds were prepared in medium to good yields from [(C_5_Ph_5_)Fe(CO)_2_Br] and either Grignard reagents *R*Mg*X* or butyl lithium in the presence of catalytic amounts of Pd(OAc)_2_ and TMEDA (Scheme 1).

The synthesis is based on a procedure that was described for the preparation of aryl iron com­plexes [(C_5_H_5_)Fe(CO)_2_Ar] (Yasuda *et al.*, 2008 ▸). Compounds **1** and **3** had been prepared before by reaction of [(C_5_Ph_5_)Fe(CO)_2_]_2_ with the corresponding alkyl iodides (no yields given; Kuksis *et al.*, 1996 ▸) or of [(C_5_Ph_5_)Fe(CO)_2_Br] and MeMgBr (55% yield; Connelly & Manners, 1989 ▸).

All four com­pounds, particularly phenyl com­pound **2**, showed a pale-blue fluorescence when irradiated at 365 nm.

### Crystal structures   

#### [(C_5_Ph_5_)Fe(CO)_2_Me], 1   

**Figure 1:**
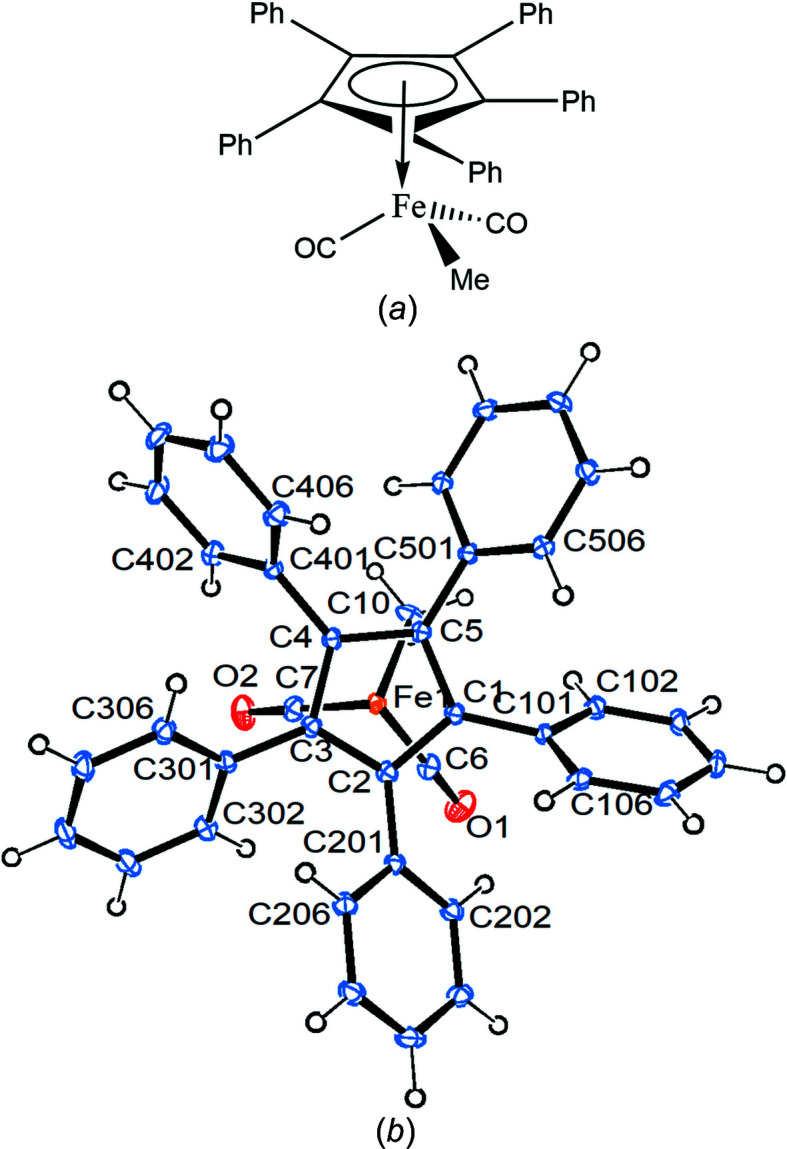
Displacement ellipsoid plot (top view) of com­pound **1**, with ellipsoids drawn at the 30% probability level.

Compound **1** crystallizes in the ortho­rhom­bic space group *Pbca* with one mol­ecule in the asymmetric unit (Fig. 1 ▸).

**Figure 2:**
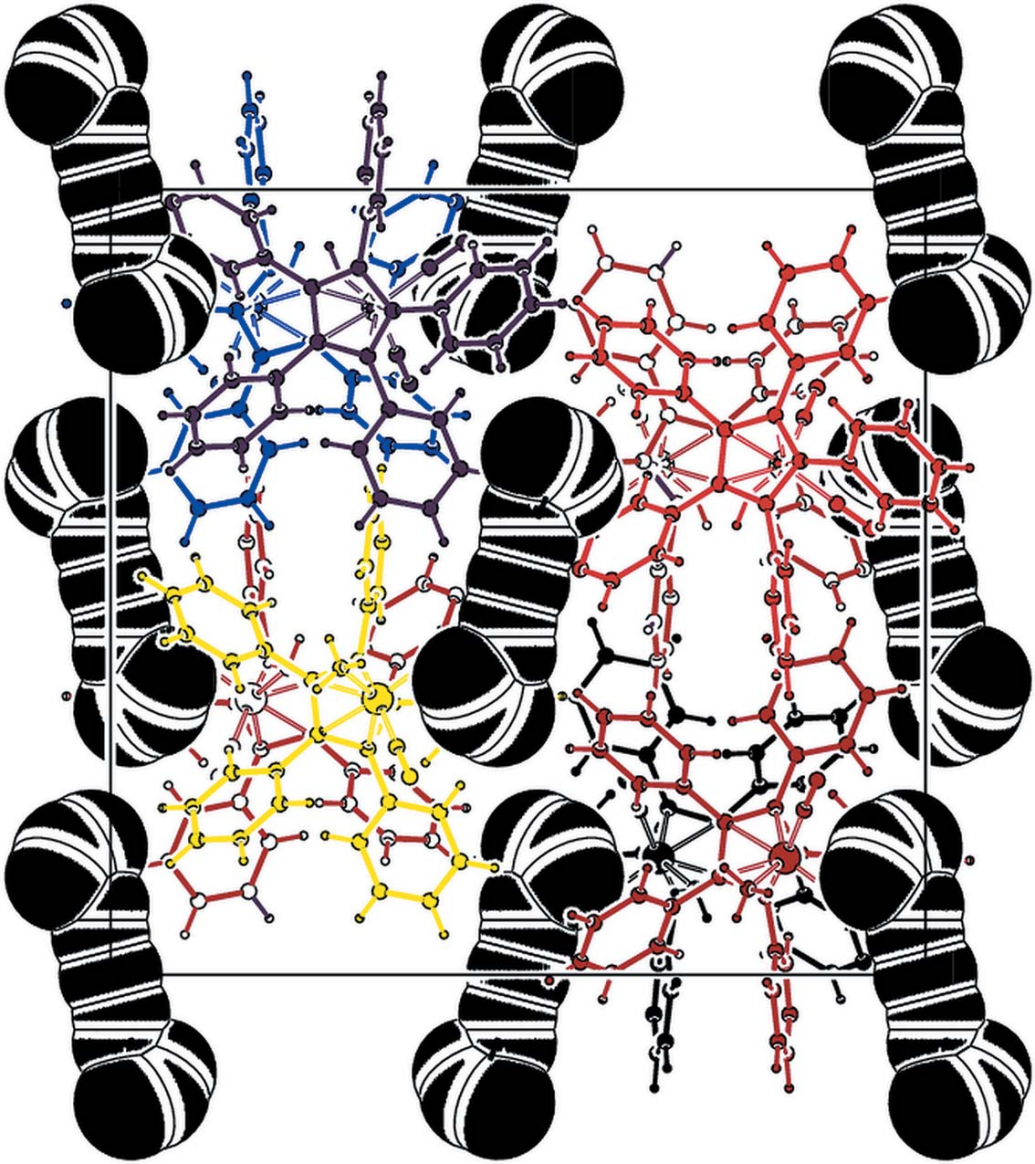
*PLATON* (Spek, 2020 ▸) cavity plot of com­pound **1**.

The Fe—C(meth­yl) bond eclipses the cyclo­penta­dienyl (Cp) C5—C501 bond, while the Fe1—C6 bond bis­ects the cyclo­penta­dienyl C1—C2 bond. All the phenyl rings are canted in the same way, as usual, with angles between the planes of the cyclo­penta­dienyl and phenyl rings ranging from 37.80 (9) to 58.66 (9)°. The Cp ring is essentially planar, with a sigpln parameter of *PLATON*, defined as:

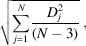

of 0.023 (Spek, 2020 ▸). Table 2 ▸ collects some important bond parameters for com­pounds **1**–**4**, together with the corresponding data from the other four published structures containing the [(C_5_Ph_5_)Fe(CO)_2_] moiety. *PLATON* analysis of the crystal structure showed that 6.3% of the volume contained solvent-accessible voids. A *PLATON* cavity plot (see Fig. 2 ▸) shows that the dumbbell-shaped voids are arranged in an fcc-type (fcc is face-centred cubic) lattice.

**Figure 3:**
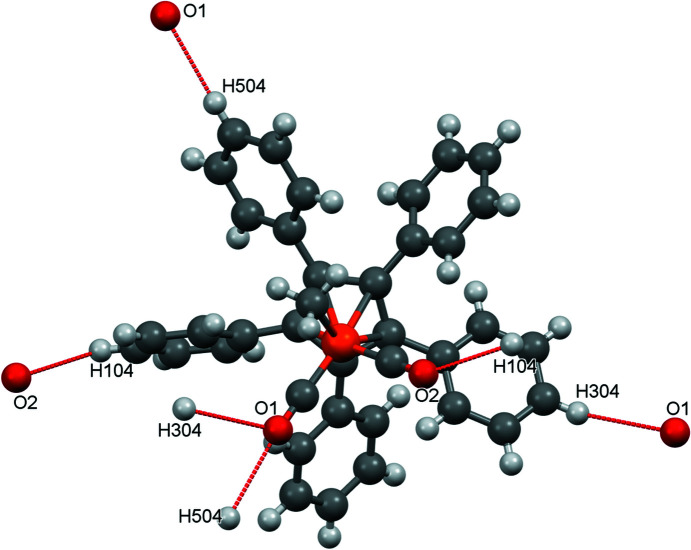
The nonclassical C—H⋯O contacts in com­pound **1**.

When looking at inter­molecular inter­actions in mercury, some ‘nonclassical’ C—H⋯O contacts [for the concept of C—H⋯O contacts, see Desiraju (2005 ▸)] appear (Fig. 3 ▸). Atom O1 accepts hydrogen bonds from H304 and H305, while atom O2 accepts a hydrogen bond from H104 (see Table 3 ▸ for the hydrogen-bond distances).

**Figure 4:**
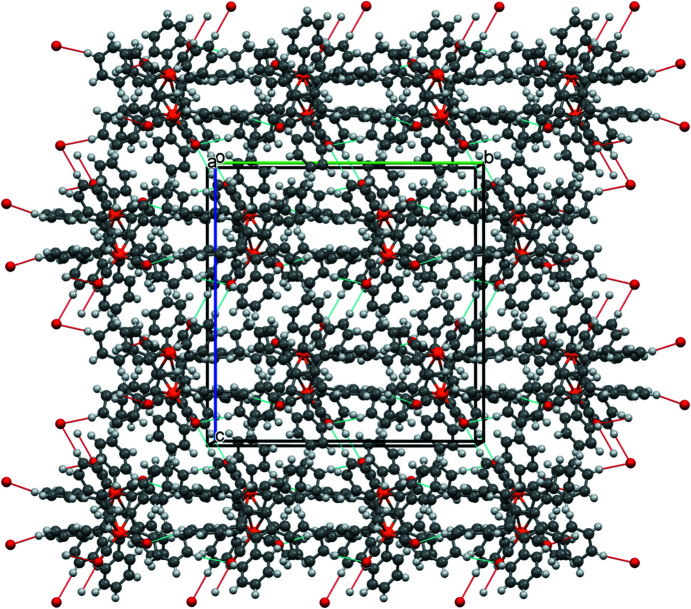
Packing diagram of com­pound **1**, viewed along the crystallographic *a* axis.

These contacts ‘join’ individual mol­ecules in all directions, leading to the packing shown in Fig. 4 ▸.

#### [(C_5_Ph_5_)Fe(CO)_2_Ph], 2   

**Figure 5:**
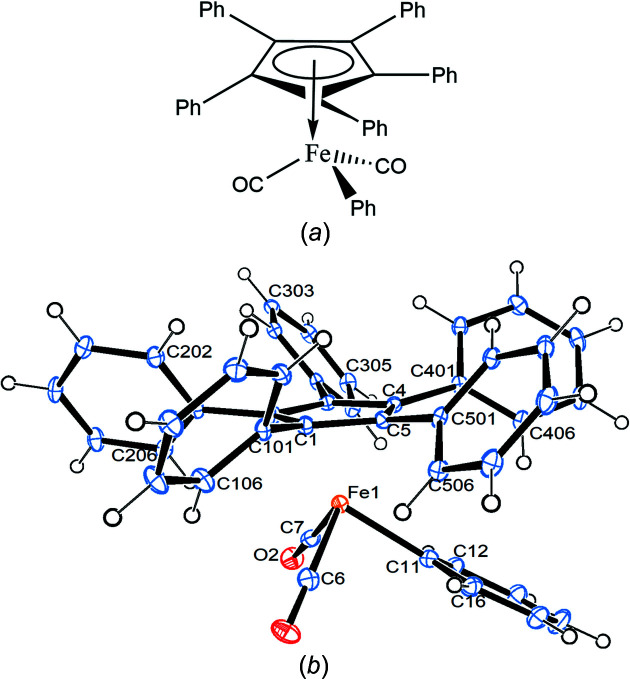
Displacement ellipsoid plot (side view) of com­pound **2**, with ellipsoids drawn at the 30% probability level.

Compound **2** crystallizes in the monoclinic space group *P*2_1_/*n* with one mol­ecule in the asymmetric unit (Fig. 5 ▸ shows a side view).

The iron–phenyl bond Fe1—C11 nearly bis­ects the cyclo­penta­dienyl C4—C5 bond. As usual, the phenyl rings exhibit a chiral propeller arrangement (but, of course, in a centrosymmetric space group like *P*2_1_/*n*, both enanti­omers are present), with inter­planar Cp–Ph angles ranging from 30.41 (12) to 59.17 (12)°. The Cp ring is essentially planar, with sigpln = 0.008. The five *ipso*-C atoms of the phenyl rings are all situated on the distal side of the Cp ring, with distances from the ring plane ranging from 0.147 (2) to 0.224 (2) Å. The σ-phenyl ring lies approximately perpendicular to the plane containing the Cp ring centroid, the Fe atom and the α-phenyl C atom, with light ‘bending’ at the α-phenyl C atom [Fe1—C11⋯C14 = 174.3 (2)°].

The closest structural ‘relative’ to **2** that can be found in the literature is CECKUS01 [(C_5_Me_5_)Fe(CO)_2_Ph] (Kalman *et al.*, 2013 ▸). There, the iron–phenyl bond length is 2.002 (2) Å. As in **2**, the σ-phenyl ring is oriented perpendicular to the plane defined by the Cp centroid, the Fe atom and the α-phenyl C atom, with a slight bend at the α-phenyl C atom.

**Figure 6:**
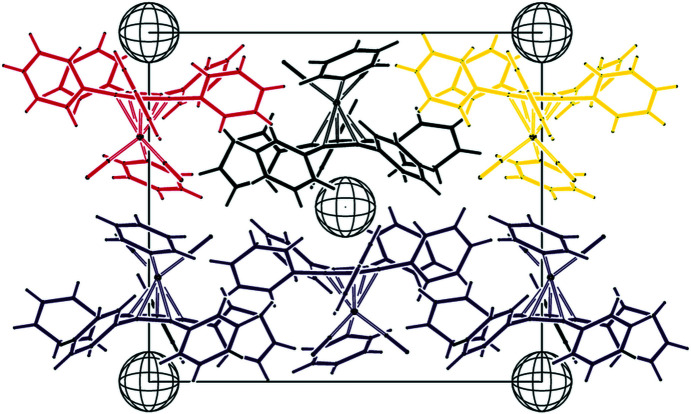
*PLATON* (Spek, 2020 ▸) cavity plot of com­pound **2**.

A *PLATON* (Spek, 2020 ▸) analysis of the crystal structure shows solvent-accessible voids of only 22 Å^3^ (0.7%). A cavity plot (Fig. 6 ▸) shows a ‘body-centred’ arrangement of these small spherical voids (radius 1.28 Å each), that appear to be ‘sandwiched’ by two cyclo­penta­dienyl rings.

**Figure 7:**
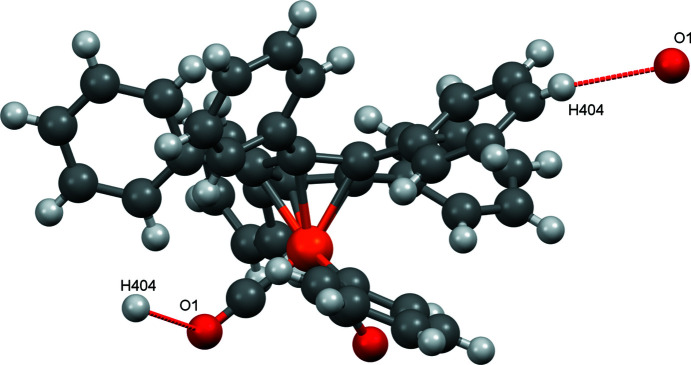
The nonclassical C—H⋯O contacts in com­pound **2**.

**Figure 8:**
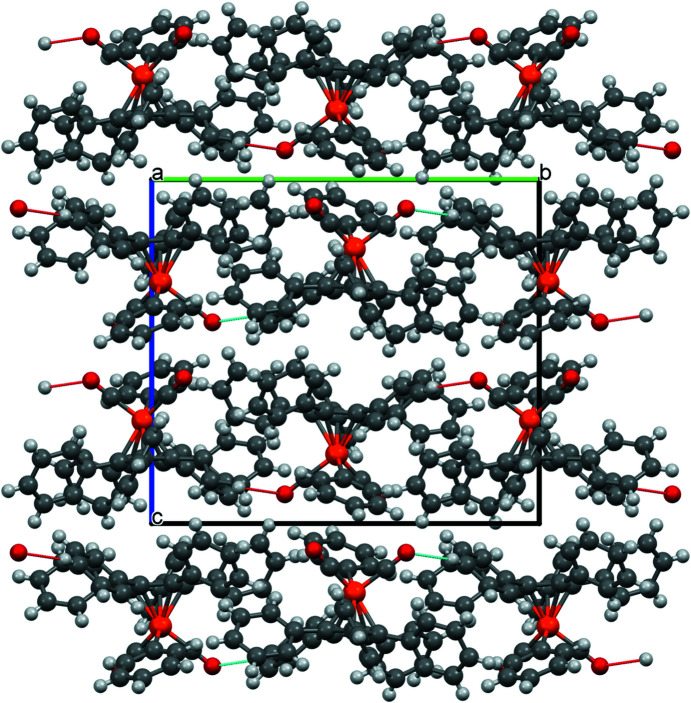
Packing diagram of com­pound **2**, viewed along the crystallographic *a* direction.

As observed for com­pound **1**, there are also C—H⋯O contacts in com­pound **2** (Fig. 7 ▸). However, here only atom O1 is involved in a contact with H404. The individual mol­ecules are ‘aligned’ by these contacts in the *a* and *b* directions (Fig. 8 ▸).

#### [(C_5_Ph_5_)Fe(CO)_2_iPr], 3   

**Figure 9:**
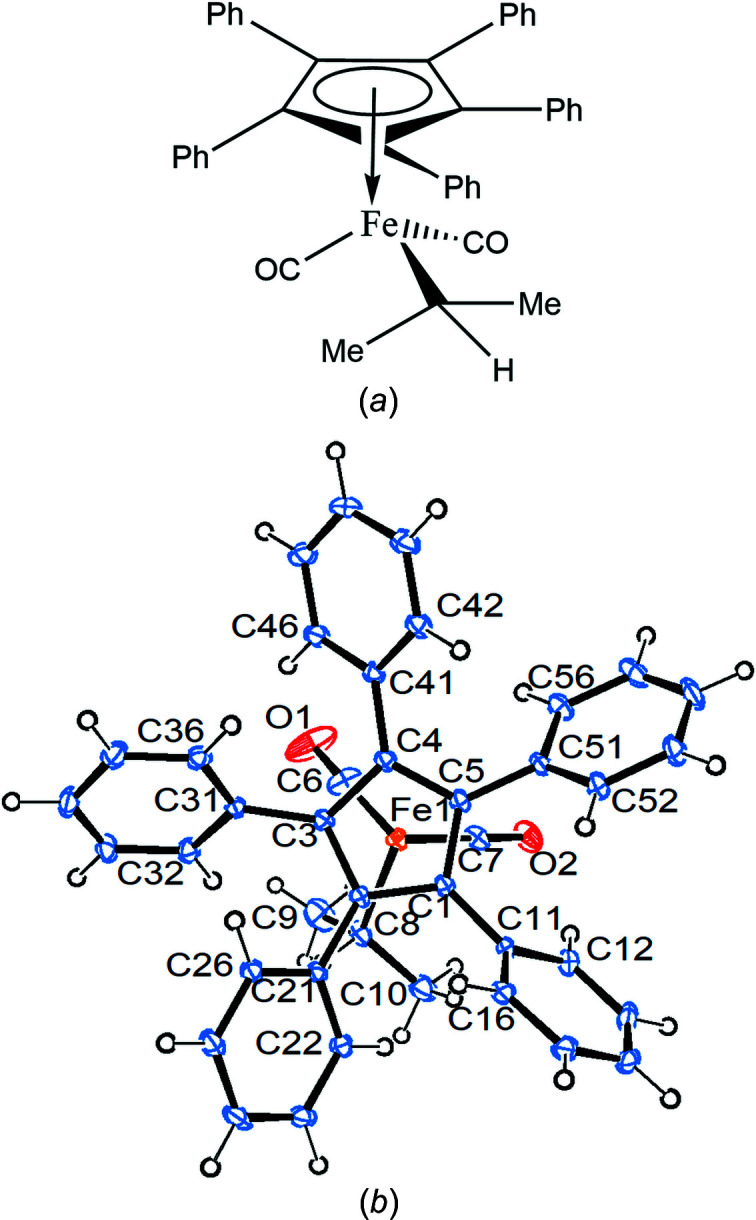
Displacement ellipsoid plot (top view) of com­pound **3**, with ellipsoids drawn at the 30% probability level.

Compound **3** also crystallizes in the monoclinic space group *P*2_1_/*n* with one mol­ecule in the asymmetric unit (Fig. 9 ▸ shows a top view).

**Figure 10:**
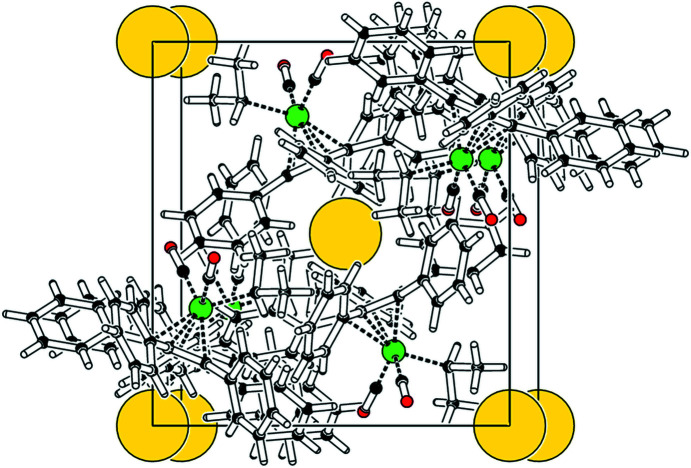
*PLATON* (Spek, 2020 ▸) cavity plot of com­pound **3**.

The iron–isopropyl bond Fe—C8 eclipses the exocyclic cyclo­penta­dienyl C2—C21 bond, while the Fe—C6O1 bond bis­ects the cyclo­penta­dienyl C3—C4 bond. Again, all the phenyl rings show a paddle-wheel orientation, with inter­planar angles ranging from 43.72 (8) and 60.94 (8)°. The cyclo­penta­dienyl ring deviates slightly from planarity, with a sigpln parameter of 0.030. All the phenyl-ring *ipso*-C atoms are located on the distal side of the Cp ring, with distances from the plane ranging from 0.056 (1) to 0.279 (1) Å. *PLATON* (Spek, 2020 ▸) analysis of the crystal structure shows solvent-accessible voids of only 18 Å^3^ (0.6%). A cavity plot (Fig. 10 ▸) again shows a body-centred arrangement of the small spherical voids (radius 1.26 Å).

**Figure 11:**
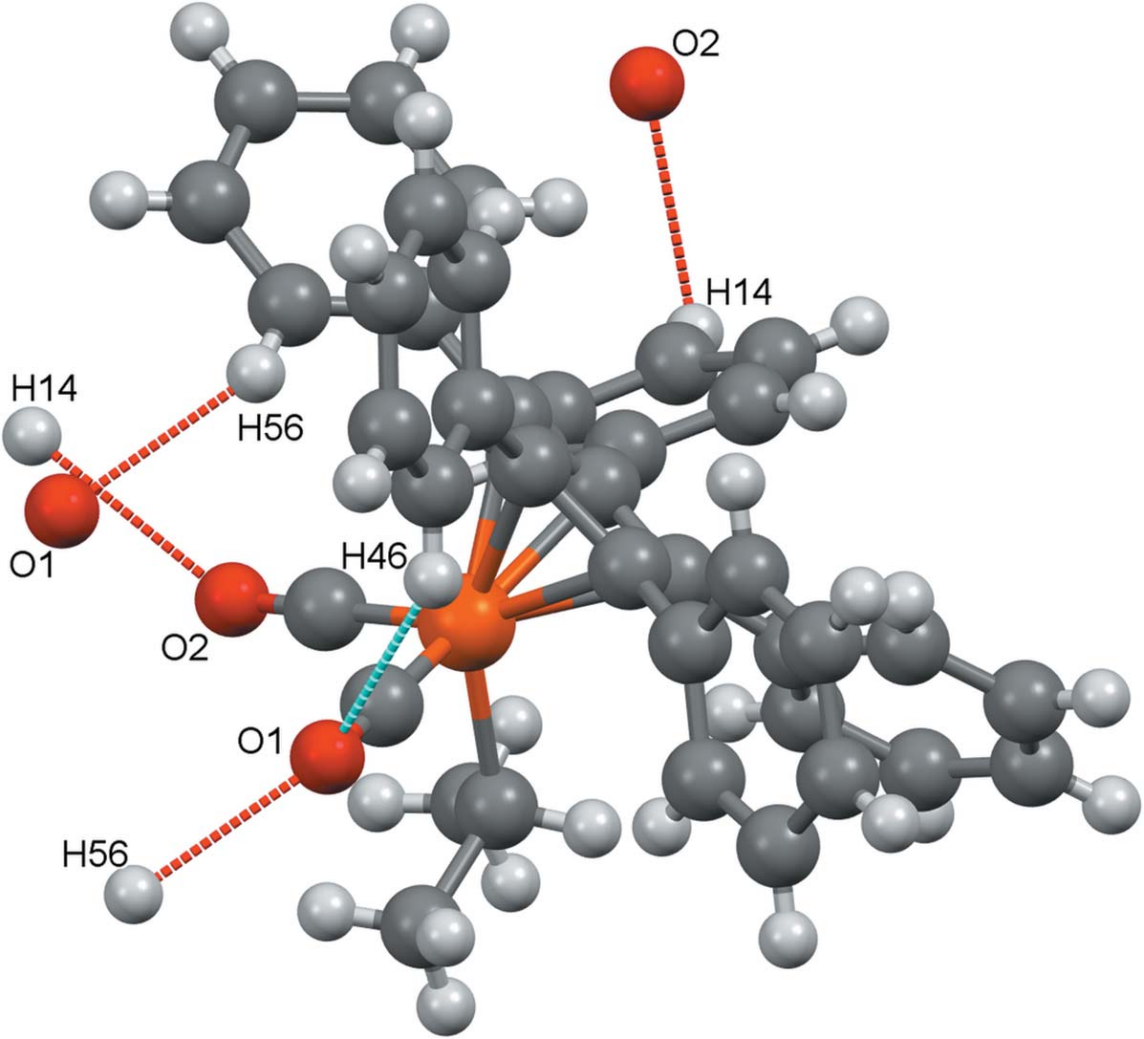
The nonclassical C—H⋯O contacts in com­pound **3**.

**Figure 12:**
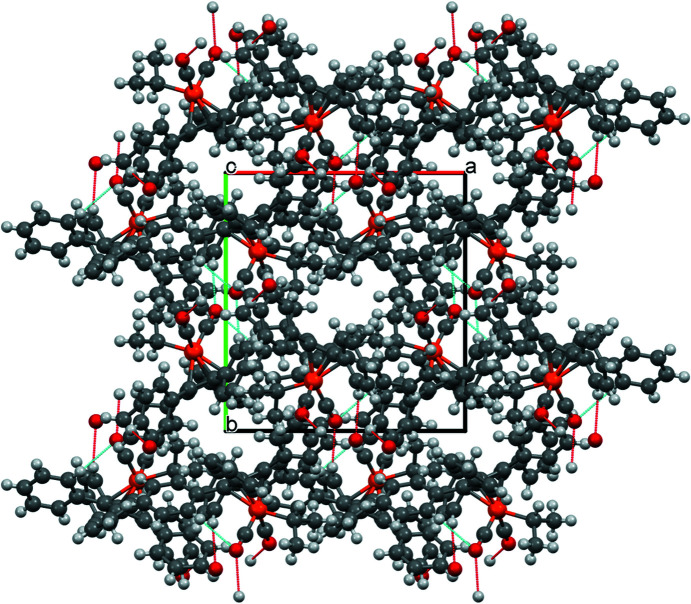
Packing diagram of com­pound **3**, viewed along the crystallographic *c* direction.

There are intra- and inter­molecular C—H⋯O contacts involving both carbonyl O atoms (Fig. 11 ▸). By means of these contacts, the individual mol­ecules are connected in all directions (Fig. 12 ▸).

#### [(C_5_Ph_5_)Fe(CO)_2_Bu], 4   

**Figure 13:**
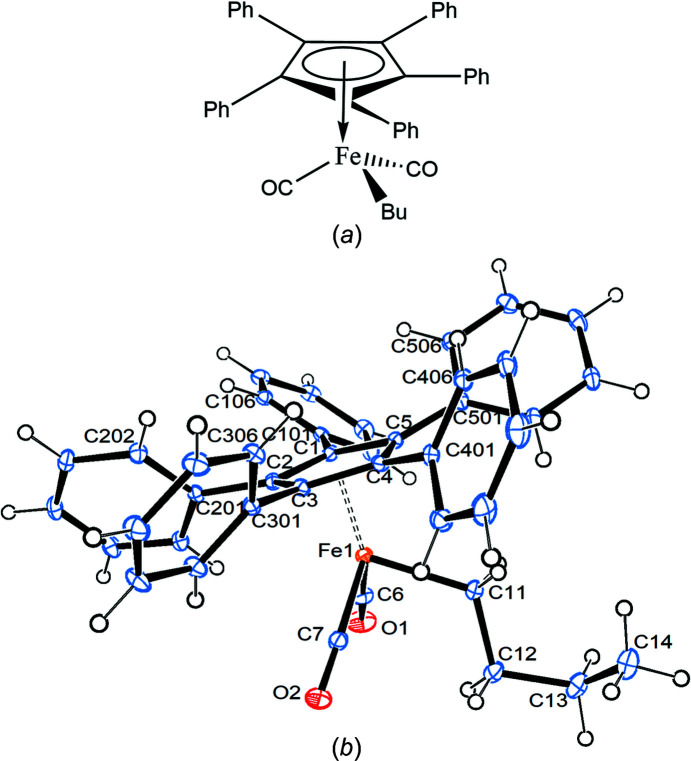
Displacement ellipsoid plot (side view) of com­pound **1**, with ellipsoids drawn at the 30% probability level.

Compound **4** also crystallizes in the monoclinic space group *P*2_1_/*n*, with one mol­ecule in the asymmetric unit (Fig. 13 ▸ shows a side view).

The iron–butyl bond bis­ects the cyclo­penta­dienyl C4—C5 bond, while both iron–carbonyl bonds, Fe—C6 and Fe—C7, eclipse the exocyclic cyclo­penta­dienyl C1—C101 and C3—C301 bonds, respectively. The C_α_—C_β_ bond of the butyl group only deviates slightly from the plane bis­ecting the Fe(CO)_2_ unit. All the phenyl rings adopt a paddle-wheel orientation, with inter­planar angles ranging from 42.85 (8) to 59.68 (7)°. The Cp ring is planar, with a sigpln parameter of 0.017. All phenyl *ipso*-C atoms are located on the distal side of the Cp ring, with distances from the plane ranging from 0.098 (1) to 0.237 (1) Å. A *PLATON* analysis (Spek, 2020 ▸) of the crystal structure shows essentially no solvent-accessible voids.

The closest structural ‘relative’ of **4** that can be found in the literature is [(C_5_Me_5_)Fe(CO)_2_(*n*-C_5_H_11_)] (CSD refcode HOZ­WIC; Hill *et al.*, 1999 ▸). There, the Fe—C_α_(alk­yl) bond has a length of 2.069 (10) Å. Similar to **4**, the C_α_—C_β_ bond of the pentyl moiety bis­ects the Fe(CO)_2_ moiety, but in contrast to **4**, all the C—C bonds of the alkyl group are in a *transoid* orientation.

**Figure 14:**
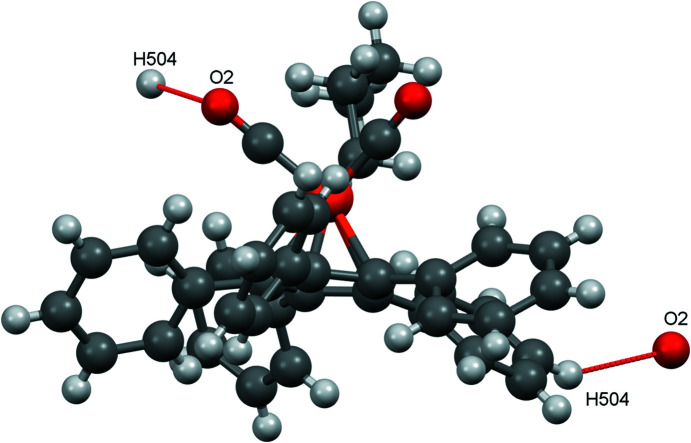
The nonclassical C—H⋯O contacts in com­pound **4**.

**Figure 15:**
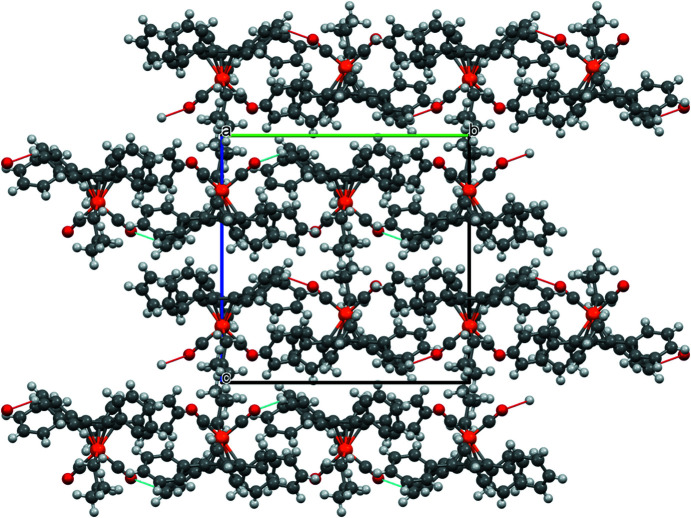
Packing diagram of com­pound **4**, viewed along the crystallographic *a* direction.

As with the other structures reported here, there are also C—H⋯O contacts in com­pound **4**, but only atom O2 is involved (see Fig. 14 ▸). The individual mol­ecules are connected in the *a* and *b* directions *via* these contacts (Fig. 15 ▸).

### Comparison of the structures of 1–4 with each other and with some other [C_5_Ph_5_] com­plexes   

Table 2 ▸ collects some important bond parameters of penta­phenyl­cyclo­penta­dienyl com­plexes, including the IR carbonyl stretching frequencies. The Fe—centroid distances (Fe—Ct) fall into three groups. The shortest bond can be found in anionic [(C_5_Ph_5_)Fe(CO)_2_][PPN] (CSD refcode PUY­DES; Carter *et al.*, 2002 ▸), with a value of 1.715 Å; a medium bond length of *ca* 1.74 Å is formed by **1** and the cationoid [(C_5_Ph_5_)Fe(CO)_2_Br] (SIRMIP; Field *et al.*, 1989 ▸), [(C_5_Ph_5_)Fe(CO)_2_(FBF_3_)] (MARFET; Hemming *et al.*, 2018 ▸) and [(C_5_Ph_5_)Fe(CO)_2_(H_2_O)]BF_4_ (MARFIX; Hemming *et al.*, 2018 ▸); and the longest bond of *ca* 1.76 Å is found for **2**–**4**. The C—O bond lengths of the metal carbonyls can also be divided in three groups. The shortest C—O bonds are found for the bromide com­plex, with a value of 1.07 (3) Å, an inter­mediate bond of 1.14 (1) Å is found for **1**–**4** and the tetra­fluoro­borate and aqua com­plexes, and the longest bond of 1.185 (3) Å is found for the anionic com­plex. This parallels the information obtained from the IR carbonyl frequencies: the highest ν(CO) value is observed for the bromide com­plex and the lowest frequencies are obtained for the anionic com­plex. Considering bond strengths, apparently the strongest metal–Cp bond and the strongest back donation to the carbonyl ligands is found for the anionic com­plex, which is not unexpected. In addition, the relative order of the C—O bond lengths (shorter/stronger for the cationoid com­plexes in com­parison with the more ‘neutral’ com­plexes) is in agreement with generally accepted bonding concepts. The only deviation from this trend is apparently the rather high ν(CO) frequency observed for phenyl com­pound **2**, which is not paralleled in the crystal structure C—O bond length. However, the relative order of the Fe—Ct distances is less dictated by electronic than by steric requirements. This is also reflected in the metal–carbon bond lengths (Fe—C_*R*_) to the alkyl or aryl residues. While the relatively short Fe—C(phen­yl) bond might indicate some back donation into the aromatic ring system [com­pare the same tendency in the pair Cp*Fe(CO)_2_C_5_H_11_/C_6_H_5_], the other Fe—C_*R*_ bonds are ordered according to the increasing steric demand of the alkyl moiety. The deviation of the phenyl *ipso*-C atoms from the cyclo­penta­dienyl ring plane is smallest for methyl com­plex **1**, largest for phenyl com­plex **2** and inter­mediate for all the other com­pounds of Table 2 ▸. The average ‘canting’ angle is smallest for phenyl com­pound **2** and largest for the bromide com­pound. The other com­pounds can be divided into two groups: an angle of 50.4 (2)° is found for **1**, **3** and **4**, and an angle of 52.4 (10)° is found for the rest. The extrema might be explained by the large size of bromine, forcing the phenyl rings into a more perpendicular orientation with respect to the cyclo­penta­dienyl ring, and on the near perpendicular orientation of the σ-phenyl ring with respect to the plane defined by Ct—Fe—C_α_, which forces the other phenyl rings into a ‘flatter’ orientation.

When com­paring the ‘nonclassical’ C—H⋯O inter­actions, it appears that nearly always the *para* H atom of one phenyl group is involved. The only exception to this ‘rule’ occurs in com­pound **3**, where two *ortho* H atoms are also involved. The observed distances are in the range 2.43–2.70 Å (Table 3 ▸). For com­parison, such contacts are also observed in [(C_5_Ph_5_)Fe(CO)_2_Br] (2.69 Å) and in [(C_5_Ph_5_)Fe(CO)_2_(H_2_O)]BF_4_ (2.534 Å). In the anionic com­plex [PPN][(C_5_Ph_5_)Fe(CO)_2_], only C—H⋯O contacts occur with the phenyl rings of the PPN^+^ cation.

## Conclusion   

Four penta­phenyl­cyclo­penta­dienyl iron alkyl and aryl com­plexes were prepared *via* a new route and characterized by IR, NMR and UV spectroscopy, and by X-ray crystallography. The mol­ecular structures show the longest distances between the Fe atom and the cyclo­penta­dienyl ring reported so far. The Fe—C(alkyl and ar­yl) bonds and the C—O bonds are in the same ranges as found for other com­pounds of this type. All com­pounds show a pale-blue solid-state fluorescence, which has not been described before for this type of com­pound. The fact that the phenyl com­pound shows a much stronger solid-state fluorescence than the others cannot be derived from the bond parameters. Despite this, all the com­pounds obviously inter­act with light and might be of use for DNA cleavage reactions. This is, however, beyond the scope of this study.

## Supplementary Material

Crystal structure: contains datablock(s) compd-1, compd-2, compd-3, compd-4, global. DOI: 10.1107/S2053229621006057/wp3017sup1.cif

Structure factors: contains datablock(s) compd-1. DOI: 10.1107/S2053229621006057/wp3017compd-1sup2.hkl

Structure factors: contains datablock(s) compd-2. DOI: 10.1107/S2053229621006057/wp3017compd-2sup3.hkl

Structure factors: contains datablock(s) compd-3. DOI: 10.1107/S2053229621006057/wp3017compd-3sup4.hkl

Structure factors: contains datablock(s) compd-4. DOI: 10.1107/S2053229621006057/wp3017compd-4sup5.hkl

CCDC references: 2089242, 2089241, 2089240, 2089239

## Figures and Tables

**Table 1:** Experimental details Experiments were carried out with Mo *K*α radiation using a Bruker D8 Venture diffractometer. Absorption was corrected for by multi-scan methods (*SADABS*; Krause *et al.*, 2015 ▸). H-atom parameters were constrained.

	**1**	**2**	**3**	**4**
Crystal data				
Chemical formula	[Fe(CH_3_)(C_35_H_25_)(CO)_2_]	[Fe(C_6_H_5_)(C_35_H_25_)(CO)_2_]	[Fe(C_3_H_7_)(C_35_H_25_)(CO)_2_]	[Fe(C_4_H_9_)(C_35_H_25_)(CO)_2_]
*M*_r_	572.45	634.52	600.50	614.53
Crystal system, space group	Orthorhombic, *P* *b* *c* *a*	Monoclinic, *P*2_1_/*n*	Monoclinic, *P*2_1_/*n*	Monoclinic, *P*2_1_/*n*
Temperature (K)	105	105	110	108
*a*, *b*, *c* (Å)	13.6393 (3), 20.4360 (5), 21.1978 (5)	12.1860 (4), 16.9411 (6), 15.0691 (6)	12.5488 (7), 13.5046 (7), 18.0119 (11)	12.1141 (4), 16.0945 (5), 16.1650 (5)
α, β, γ (°)	90, 90, 90	90, 93.320 (1), 90	90, 93.208 (2), 90	90, 95.706 (1), 90
*V* (Å^3^)	5908.5 (2)	3105.71 (19)	3047.6 (3)	3136.08 (17)
*Z*	8	4	4	4
μ (mm^−1^)	0.54	0.52	0.53	0.52
Crystal size (mm)	0.06 × 0.05 × 0.04	0.08 × 0.02 × 0.02	0.06 × 0.04 × 0.03	0.08 × 0.05 × 0.04

Data collection				
*T*_min_, *T*_max_	0.718, 0.746	0.669, 0.745	0.719, 0.746	0.832, 0.862
No. of measured, independent and observed [*I* > 2σ(*I*)] reflections	61228, 6765, 5439	31592, 6352, 5025	52953, 6732, 5995	55494, 7202, 6246
*R*_int_	0.053	0.050	0.031	0.041
(sin θ/λ)_max_ (Å^−1^)	0.649	0.625	0.641	0.650

Refinement				
*R*[*F* ^2^ > 2σ(*F* ^2^)], *wR*(*F* ^2^), *S*	0.035, 0.090, 1.06	0.041, 0.088, 1.04	0.032, 0.088, 1.06	0.033, 0.090, 1.05
No. of reflections	6765	6352	6732	7202
No. of parameters	371	415	390	398
Δρ_max_, Δρ_min_ (e Å^−3^)	0.35, −0.49	0.32, −0.41	0.36, −0.36	0.37, −0.47

Computer programs: *APEX2* (Bruker, 2011 ▸), *SAINT* (Bruker, 2011 ▸), *SHELXT2018* (Sheldrick, 2015*a* ▸), *SHELXL2018* (Sheldrick, 2015*b* ▸), *ORTEP-3 for Windows* (Farrugia, 2012 ▸), *Mercury* (Macrae *et al.*, 2020 ▸) and *WinGX* (Farrugia, 2012 ▸).

**Table 2:** Comparison of important bond parameters (Å, °) of com­pounds **1**–**4** and some related structures from the CSD

	**1**	**2**	**3**	**4**	SIRMIP	MARFET	MARFIX	PUYDES	HOZWIC	CECKUS01
Fe—Ct (Å)	1.7403 (8)	1.7625 (10)	1.7586 (7)	1.7603 (7)	1.738 (5)	1.7464 (10)	1.7360 (12)/	1.715 (3)	1.730	1.730
							1.7306 (12)			
Fe—C_α_(*R*) (Å)	2.073 (2)	2.022 (2)	2.1188 (17)	2.0810 (16)	n.a.	n.a.	n.a.	n.a.	2.069 (10)	2.002 (2)
Fe—C(CO) (Å)	1.751 (2)	1.755 (2)	1.745 (2)	1.755 (2)	1.812 (5)		1.803 (3)	1.714 (6)	1.739 (11)	1.756 (1)
	1.754 (2)	1.760 (2)	1.756 (2)	1.755 (2)	1.786 (5)		1.813 (3)	1.715 (6)	1.751 (11)	
C—O (Å)	1.149 (2)	1.152 (3)	1.145 (3)	1.150 (1)	1.052 (6)	1.133 (3)	1.131 (4)/	1.182 (7)	1.153 (13)	1.151 (2)
	1.136 (2)	1.148 (3)	1.148 (2)	1.148 (2)	1.097 (6)	1.137 (4)	1.126 (4)	1.187 (7)	1.139 (14)	1.151 (2)
							1.137 (3)/			
							1.132 (4)			
IR (ν_CO_, cm^−1^)	1993, 1941	2009, 1968	1991, 1939	1993, 1939	2033, 1993*			1872, 1806*	1987, 1933*	1994, 1937*
(C_*i*,Ph_—Cp)_av_ (Å)	0.122	0.188	0.144	0.160	0.15	0.147	0.144/0.145		n.a.	n.a.
(Cp–Ph)_av_ (°)	50.3	48.5	50.6	50.2	58.7	52.3	51.8/51.4	53.4	n.a.	n.a.
[C_α_—Fe—Ct—C_Cp_]_min_	9.35	26.46	5.13	32.19	n.a.	n.a.	n.a.	n.a.	1.44	0.0
Ct—Fe—C_α_—C_β_ (°)	n.a.	95.8	141.1	167.4	n.a.	n.a.	n.a.	n.a.	179.9	87.5
		79.8	91.8							

CSD refcodes: SIRMIP is [(C_5_Ph_5_)Fe(CO)_2_Br] (Field *et al.*, 1989 ▸), MARFET is [(C_5_Ph_5_)Fe(CO)_2_FBF_3_] (Hemming *et al.*, 2018 ▸), MARFIX is [(C_5_Ph_5_)Fe(CO)_2_(H_2_O)]BF_4_ (Hemming *et al.*, 2018 ▸), PUYDES is [PPN][(C_5_Ph_5_)Fe(CO)_2_] (Carter *et al.*, 2002 ▸), HOZWIC is [(C_5_Me_5_)Fe(CO)_2_C_5_H_11_] (Hill *et al.*, 1999 ▸) and CECKUS01 is [(C_5_Me_5_)Fe(CO)_2_Ph] (Kalman *et al.*, 2013 ▸). Notes: Ct is the centroid of the cyclo­penta­dienyl ring. (C_*i*,Ph_—Cp)_av_ is the average distance of the phenyl *ipso*-C atoms from the plane of the Cp ring. (Cp–Ph)_av_ is the average dihedral angle of the five phenyl rings with respect to the plane of the Cp ring, [C_α_—Fe—Ct—C_Cp_]_min_ is the smallest torsion angle between the α-alk­yl/aryl C atom, the Fe atom, the centroid of the cyclo­penta­dienyl ring and a Cp-ring C atom. ‘n.a.’ denotes not applicable. The asterisk (*) denotes solution spectra.

**Table 3:** Nonclassical C—H⋯O contacts in **1**–**4**

Compound	Atom pair	Distance (Å)	Symmetry code
**1**	H504⋯O1	2.597	−*x* + {1\over 2}, −*y* + 1, *z* + {1\over 2}
	H104⋯O2	2.611	−*x* + {1\over 2}, *y* − {1\over 2}, *z*
	O1⋯H304	2.703	−*x* + {1\over 2}, *y* − {1\over 2}, *z*
**2**	O1⋯H404	2.475	−*x* + {1\over 2}, *y* − {1\over 2}, −*z* + {3\over 2}
**3**	H14⋯O2	2.694	−*x* + {1\over 2}, *y* − {1\over 2}, −*z* + {1\over 2}
	H56⋯O1	2.430	−*x* + 1, −*y* + 2, −*z* + 1
**4**	O2⋯H504	2.589	−*x* + {3\over 2}, *y* − {1\over 2}, −*z* + {3\over 2}

## References

[bb1] Argouarch, G., Grelaud, G., Roisnel, T., Humphrey, M. G. & Paul, F. (2012). *Tetrahedron Lett.* **53**, 5015–5018.

[bb2] Brégaint, P., Hamon, J.-R. & Lapinte, C. (1990). *J. Organomet. Chem.* **398**, C25—C28.

[bb3] Brégaint, P., Hamon, J.-R. & Lapinte, C. (1992). *Organometallics*, **11**, 1417–1419.

[bb4] Bruker (2011). *APEX2* and *SAINT*. Bruker AXS Inc., Madison, Wisconsin, USA.

[bb5] Carter, B. T., Castellani, M. P., Rheingold, A. L., Hwang, S., Longacre, S. E. & Richmond, M. G. (2002). *Organometallics*, **21**, 373–379.

[bb6] Connelly, N. G. & Manners, I. (1989). *J. Chem. Soc. Dalton Trans.* pp. 283–288.

[bb7] Desiraju, G. R. (2005). *Chem. Commun.* pp. 2995–3001. 10.1039/b504372g 15959566

[bb8] Farrugia, L. J. (2012). *J. Appl. Cryst.* **45**, 849–854.

[bb9] Field, L. D., Hambley, T. W., Lindall, C. M. & Masters, A. F. (1989). *Polyhedron*, **8**, 2425–2430.

[bb10] Field, L. D., Lindall, C. M., Masters, A. F. & Clentsmith, G. K. B. (2011). *Coord. Chem. Rev.* **255**, 1733–1790.

[bb11] Fukumoto, K., Kasa, M. & Nakazawa, H. (2015). *Inorg. Chim. Acta*, **431**, 219–221.

[bb12] Groom, C. R., Bruno, I. J., Lightfoot, M. P. & Ward, S. C. (2016). *Acta Cryst.* B**72**, 171–179. 10.1107/S2052520616003954 PMC4822653 27048719

[bb13] Hemming, E. B., Chan, B., Turner, P., Corcilius, L., Price, J. R., Gardiner, M. G., Masters, A. F. & Maschmeyer, T. (2018). *Appl. Catal. Environ.* **223**, 234–241.

[bb14] Hill, R. O., Marais, C. F., Moss, J. R. & Naidoo, K. J. (1999). *J. Organomet. Chem.* **587**, 28–37.

[bb15] Kalman, S. E., Petit, A., Gunnoe, T. B., Ess, D. H., Cundari, T. R. & Sabat, M. (2013). *Organometallics*, **32**, 1797–1806.

[bb16] Klein-Heßling, C., Blockhaus, T. & Sünkel, K. (2021). *J. Organomet. Chem.* **943**, 121833.

[bb17] Krause, L., Herbst-Irmer, R., Sheldrick, G. M. & Stalke, D. (2015). *J. Appl. Cryst.* **48**, 3–10. 10.1107/S1600576714022985 PMC4453166 26089746

[bb18] Kuksis, I. & Baird, M. C. (1994). *Organometallics*, **13**, 1551–1553.

[bb19] Kuksis, I., Kovács, I., Baird, M. C. & Preston, K. F. (1996). *Organometallics*, **15**, 4991–5002.

[bb20] Macrae, C. F., Sovago, I., Cottrell, S. J., Galek, P. T. A., McCabe, P., Pidcock, E., Platings, M., Shields, G. P., Stevens, J. S., Towler, M. & Wood, P. A. (2020). *J. Appl. Cryst.* **53**, 226–235. 10.1107/S1600576719014092 PMC6998782 32047413

[bb21] McVey, S. M. & Pauson, P. L. (1965). *J. Chem. Soc.* pp. 4312–4318.

[bb22] Mohler, D. L., Barnhardt, E. K. & Hurley, A. L. (2002). *J. Org. Chem.* **67**, 4982–4984. 10.1021/jo0257290 12098319

[bb23] Mohler, D. L. & Shell, T. A. (2005). *Bioorg. Med. Chem. Lett*. **15**, 4785–4788. 10.1016/j.bmcl.2005.06.102 16115764

[bb24] Pannell, K. H. & Sharma, H. K. (2010). *Organometallics*, **29**, 4741–4745. 10.1021/om901114q PMC2840652 20305775

[bb25] Ruble, J. C., Latham, H. A. & Fu, G. C. (1997). *J. Am. Chem. Soc.* **119**, 1492–1493.

[bb26] Schulte, Y., Weinert, H., Wölper, C. & Schulz, S. (2020). *Organometallics*, **39**, 206–216.

[bb27] Sheldrick, G. M. (2015*a*). *Acta Cryst.* A**71**, 3–8.

[bb28] Sheldrick, G. M. (2015*b*). *Acta Cryst.* C**71**, 3–8.

[bb29] Spek, A. L. (2020). *Acta Cryst.* E**76**, 1–11. 10.1107/S2056989019016244 PMC6944088 31921444

[bb30] Sünkel, K., Weigand, S., Hoffmann, A., Blomeyer, S., Reuter, C. G., Vishnevskiy, Y. V. & Mitzel, N. W. (2015). *J. Am. Chem. Soc.* **137**, 126–129. 10.1021/ja511588p 25531826

[bb31] Yasuda, S., Yorimitsu, H. & Oshima, K. (2008). *Organometallics*, **27**, 4025–4027.

